# Prenatal diesel exhaust exposure alters hippocampal synaptic plasticity in offspring

**DOI:** 10.18632/aging.205592

**Published:** 2024-03-01

**Authors:** Shali Yu, Ziyang Zhang, Ziyu Qin, Meijun Liu, Xiaoye Zhao, Yulan Cheng, Peng Xue, Xiaoke Wang, Lin Chen, Qiyun Wu, Linling Ju, Juan Tang

**Affiliations:** 1 Department of Occupational Medicine and Environmental Toxicology, Nantong Key Laboratory of Environmental Toxicology, School of Public Health, Nantong University, Nantong 226019, China; 2 Institute of Liver Diseases, Nantong Third People’s Hospital, Affiliated Nantong Hospital 3 of Nantong Hospital 3 of Nantong University, Nantong 226006, China

**Keywords:** diesel exhaust particles, neurotoxicity, hippocampus, CPEB3, NMDA receptor

## Abstract

Diesel exhaust particles (DEPs) are major air pollutants emitted from automobile engines. Prenatal exposure to DEPs has been linked to neurodevelopmental and neurodegenerative diseases associated with aging. However, the specific mechanism by DEPs impair the hippocampal synaptic plasticity in the offspring remains unclear. Pregnant C57BL/6 mice were administered DEPs solution via the tail vein every other day for a total of 10 injections, then the male offsprings were studied to assess learning and memory by the Morris water maze. Additionally, protein expression in the hippocampus, including CPEB3, NMDAR (NR1, NR2A, NR2B), PKA, SYP, PSD95, and p-CREB was analyzed using Western blotting and immunohistochemistry. The alterations in the histomorphology of the hippocampus were observed in male offspring on postnatal day 7 following prenatal exposure to DEPs. Furthermore, 8-week-old male offspring exposed to DEPs during prenatal development exhibited impairments in the Morris water maze test, indicating deficits in learning and memory. Mechanistically, the findings from our study indicate that exposure to DEPs during pregnancy may alter the expression of CPEB3, SYP, PSD95, NMDAR (NR1, NR2A, and NR2B), PKA, and p-CREB in the hippocampus of both immature and mature male offspring. The results offer evidence for the role of the NMDAR/PKA/CREB and CPEB3 signaling pathway in mediating the learning and memory toxicity of DEPs in male offspring mice. The alterations in signaling pathways may contribute to the observed damage to synaptic structure and transmission function plasticity caused by DEPs. The findings hold potential for informing future safety assessments of DEPs.

## INTRODUCTION

Air pollution caused by particulate matter (PM) poses a significant global public health threat. Extensive research has established a causal relationship between ambient PM and health risks in urban areas [1, 2]. Diesel exhaust particles (DEPs) are a prominent constituent of environmental PM, with the majority of particles being emitted directly from diesel exhaust and having a diameter of less than 1mm [3]. Diesel and engine emissions of DEP represent the primary sources of airborne PM, which can persist in the atmosphere and easily enter the respiratory system upon inhalation [4]. Epidemiological and clinical studies have consistently demonstrated a strong association between DEP pollution and cardiopulmonary diseases, as well as chronic obstructive pulmonary disease [5, 6]. Furthermore, toxicology studies have provided evidence that exposure to DEPs can lead to neurotoxicity in mice [7], highlighting a potential link between exposure to environmental pollutants and neurological disorders in rodents.

Recently, increasing reports have shown that the nanoscale components of PM can access the brain and may be related to neurodegenerative diseases such as Alzheimer’s disease [8–11]. These particles have the capacity to penetrate the blood-brain barrier, and they can also carry various toxic compounds on their surface, including hydrocarbons and metals [12]. Additionally, the presence of ultrafine DEPs in the brain tissue of newborn mice, specifically in the hippocampus and cerebral cortex, indicates that exposure of pregnant mice to DEPs might have an impact on the morphology of the hippocampus and cortex, as well as on brain development in their offspring [13].

Hippocampal synaptic plasticity is essential for learning and memory processes and can be affected by environmental toxins [14–16]. In neurodegenerative conditions, it is believed that synapses deteriorate earlier than neurons, making it a potential early indicator of changes in learning and memory abilities [17]. Synaptic plasticity disorders in the hippocampus involve abnormalities in both synaptic transmission function and structural plasticity. Postsynaptic density-95 (PSD95) and synaptophysin (SYP) are commonly used markers associated with synaptic structural plasticity [18]. Phosphorylated CREB (p-CREB), the active form of the second messenger cAMP responsive element binding protein (CREB), plays a key role in regulating various neuroprotective factors and enhancing synaptic efficiency and structural plasticity [19, 20]. N-methyl-D-aspartate receptors (NMDARs), as part of the ionotropic glutamate receptor (iGluR) family, are essential for brain signaling and development. They are involved in the transmission of excitatory neurotransmission signals which play a crucial role in synaptic plasticity. NMDARs, including NMDA receptor 1 (NR1), NMDA receptor 2A (NR2A), and NMDA receptor 2B (NR2B), have complex functions and structures [21, 22]. Each subtype has its own unique characteristics, and disruptions in NMDAR-related signaling can have adverse effects on advanced cognitive functions such as learning and memory in various neurological and psychiatric disorders. Consequently, their mechanisms have been extensively investigated for decades [23–25]. Long-term memory maintenance is known to require the synthesis of new proteins and is closely associated with translational regulation. The cytoplasmic polyadenylation element-binding protein 3 (CPEB3) plays a critical role in establishing synaptic plasticity and memory. It regulates the translation of several proteins, including NMDARs (NR1, NR2A, and NR2B), PSD95, and SYP, in the hippocampus [26].

In this experiment, we investigated the neurotoxic effects of prenatal exposure to DEPs on learning and memory in male offspring. We established a mouse model where pregnant mice were exposed to different doses of DEPs. We measured the levels of synaptic plasticity-associated proteins and the expression of PKA, CPEB3, p-CREB, and CREB proteins in the hippocampus of male offspring at postnatal day 7 (PND7) and in adulthood. Additionally, we evaluated the cognitive behaviors of the adult male offspring. The study was to identify the underlying mechanisms of neurotoxicity by comparing the effects on early postnatal and adult male offspring of DEP exposure throughout pregnancy.

## RESULTS

### Effects of prenatal exposure to DEPs on the body weight and organ weight of male offspring

**Figure 1:**
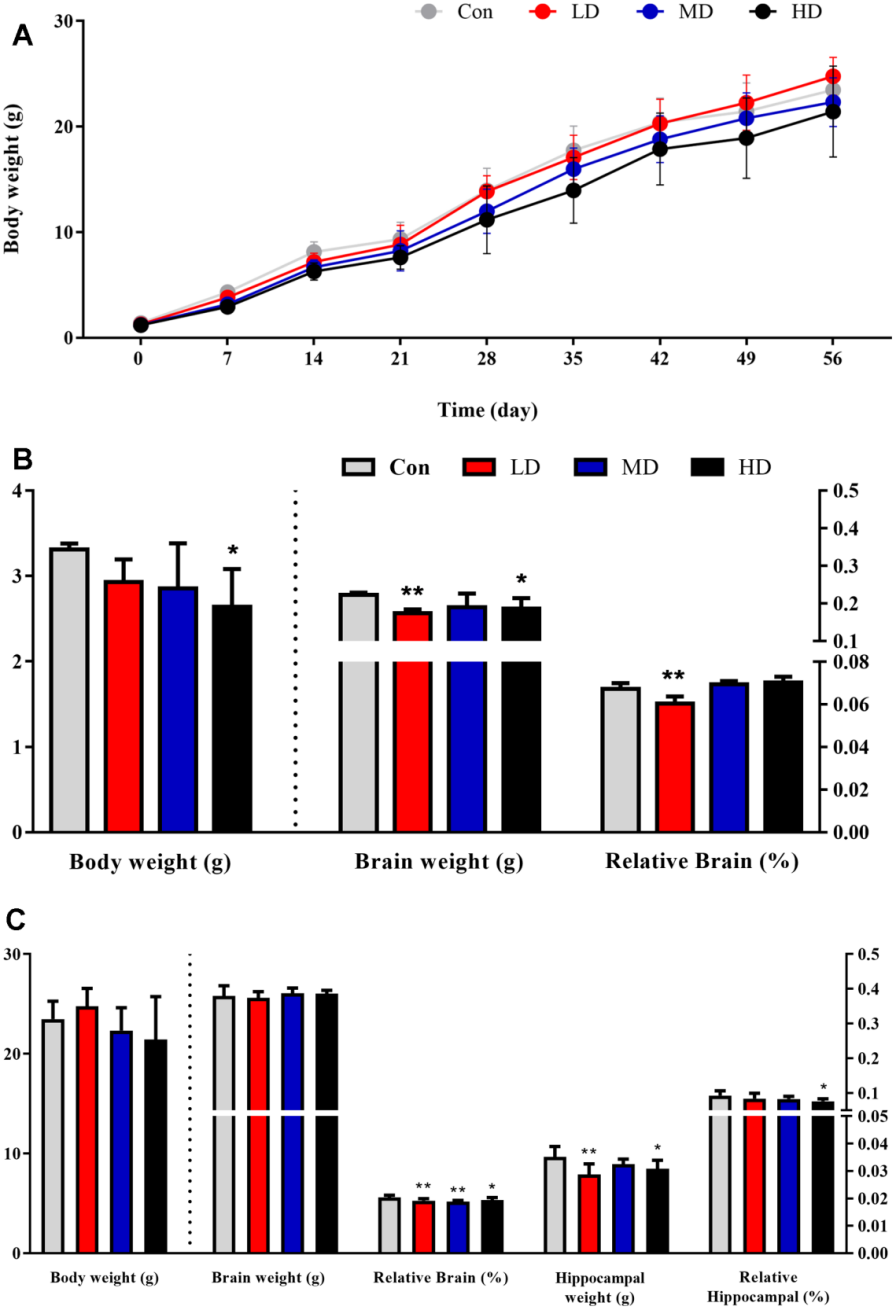
**Body weight and organ weight of male offspring after prenatal DEPs exposure.** (**A**) The body weight of male offspring. (**B**) The body weight, brain weight and relative brain of 7 days male offspring. (**C**) The body weight, brain weight, relative brain, hippocampal weight and relative hippocampal of 56 days male offspring. The values shown are the mean ± SD. Compared to control; * *p* < 0.05, ** *p* < 0.01. N0,7day (Con: Control group; LD: low dose group; MD: medium dose group) =22, N0, 7 day (HD:High dose group)=12, N14-56day (each group)=12.

The male offspring mean body weight, but not brain weight, was found to be affected by prenatal exposure to DEPs from birth to adulthood (Figure 1). During the first 6 weeks, the body weight of the control group’s offspring remained higher than the other groups. Starting from week 7, the low-dose group’s offspring exhibited faster growth compared to the control group. Nevertheless, the average body weight of the medium and high-dose group’s offspring remained lower than that of the control group on postnatal day 56 (PND56). However, these differences were not statistically significant due to individual variations within the groups (Figure 1A, 1C). Interestingly, compared to the control group, the mean body weight and brain weight of male offspring in the high-dose group were significantly lower on postnatal day 7 (PND7), both brain weight and the brain-to-body weight ratio were significantly lower in the low-dose groups (Figure 1B). This indicates that prenatal exposure to DEPs may impair the growth and development of the offspring.

### Histological effects of prenatal DEPs exposure on PND7 male offspring

**Figure 2:**
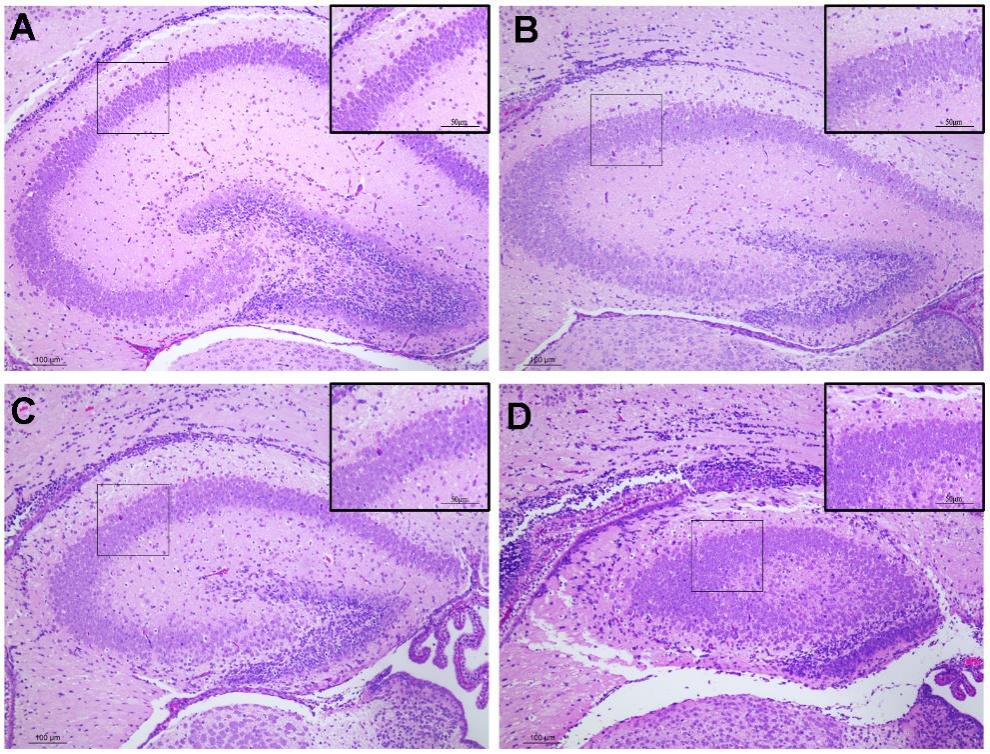
**Histopathological changes in the hippocampus tissues of male offspring mice following pregnancy exposure to DEPs through hematoxylin and eosin (H&E) staining. 100 or 200 × magnification.** (**A**) Control group. The morphology of hippocampus tissues was normal. (**B**–**D**) DEPs (0.235, 0.47 and 0.94 μg/mouse) treatment groups. Scale bar: 100 μm. N=4.

We assessed the histology of the hippocampus in PND7 male offspring using H&E staining to investigate the impact of prenatal exposure to DEPs on the hippocampus of early postnatal male mice. In the control group, hippocampal neurons were organized into dense neuronal layers, exhibiting a normal structure and no signs of damage to the hippocampus (Figure 2A). However, in the DEPs treatment groups, the arrangement of hippocampal neuron cells appeared disordered, accompanied by cell swelling, slight cytoplasm staining, cytoplasmic vacuolization, intense nuclear staining, and nuclear pyknosis (Figure 2B–2D). These findings suggest that exposure to DEPs throughout pregnancy significantly influences the development and morphology of the hippocampus in male offspring mice.

### The impact of exposure to DEPs throughout pregnancy on cognitive function of mature male offspring

**Figure 3:**
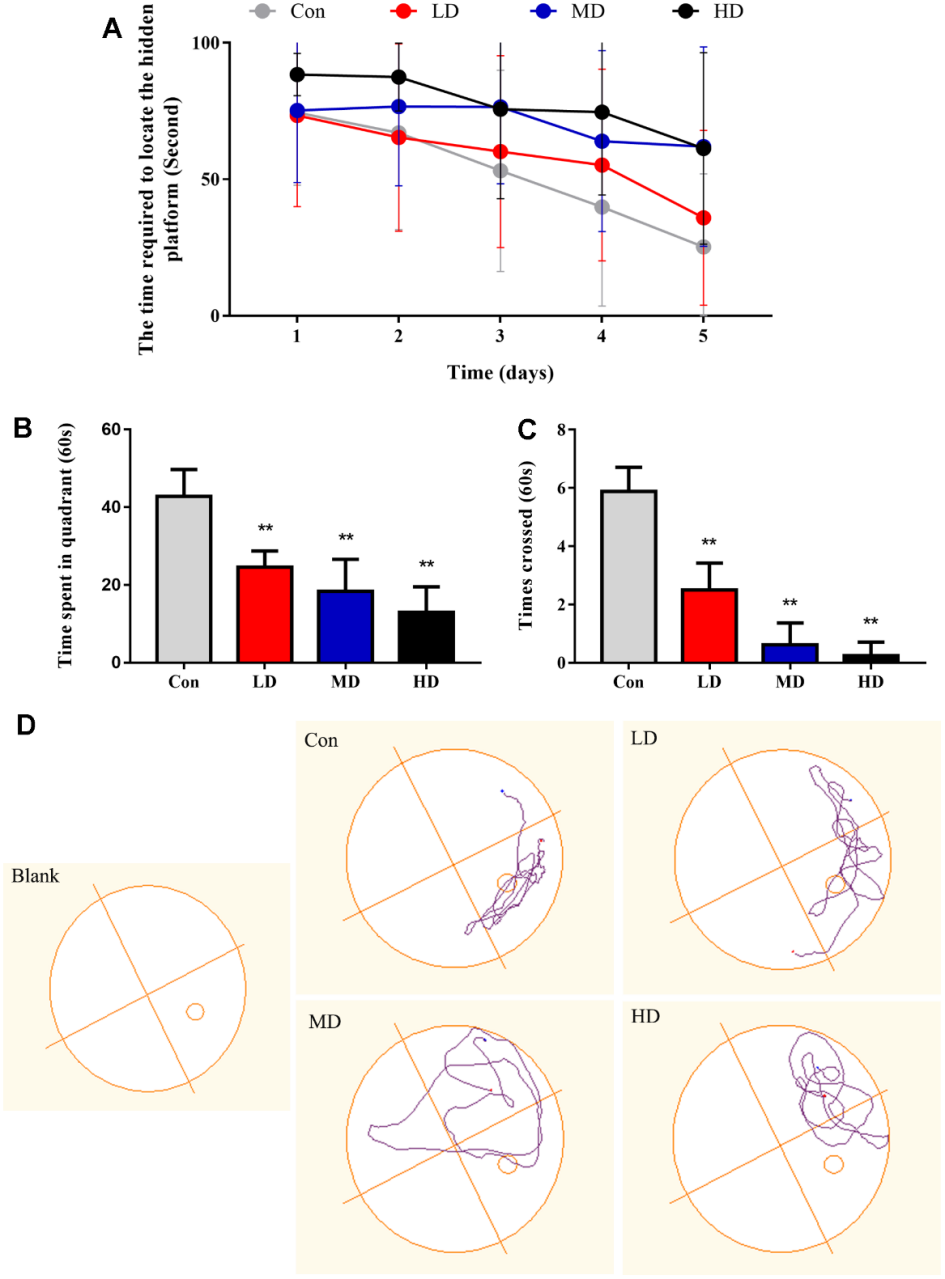
**Performance of the different groups of mice in the Morris water maze test.** (**A**) The time required to locate the hidden platform in the water maze during the learning stage. (**B**) The time elapsed in the correct quarter of the water maze during the probe trial. (**C**) Number of platform crossings in the four quadrants of the Morris maze during the probe trial. (**D**) Representative spatial and nonspatial probe trial tracings of four subjects. In the spatial version, the placement of the quadrant remained constant over trials. Con: Control group; LD: low dose group; MD: medium dose group; HD: high dose group. The values shown are the mean ± SD (n_each group_=12), Compared to control; ** *p* < 0.01.

The spatial learning and memory abilities were employed to assess by the Morris water maze test, which reflected the cognitive function of mature male offspring (PND49-PND56) following exposure to DEPs throughout pregnancy (Figure 3D). The results indicated that the escape latency of the trained mice decreased as the training days progressed, suggesting that all groups of mice possessed a learning ability (Figure 3A). Nevertheless, the differences between each group were dependent on their respective treatments. Specifically, apart from the high-dose (HD) group, there were no significant differences in the escape latency among the other three groups on the first day of spatial training. However, from the second to the fifth day of training, the DEPs treatment groups exhibited longer escape latencies compared to the control group. After the completion of the spatial navigation test, the platform was removed, and the mice were allowed to choose one of the original entry points into the water. The number of mice crossing the target quadrant within a span of 60 seconds was recorded. The results indicated that the average frequency of crossing the platform of the DEPs treatment group was significantly lower than that of the control group (P<0.01) (Figure 3C). Furthermore, the male offspring exposed to DEPs spent less time in the target quadrant compared to the control group (P<0.01) (Figure 3B). Collectively, these findings suggest that exposure to DEPs throughout pregnancy impairs the cognitive abilities of male offspring.

### The influence of exposure to DEPs throughout pregnancy on the hippocampal synaptic proteins expression, PKA/CREB and CPEB3 activation in PND7 offspring

**Figure 4:**
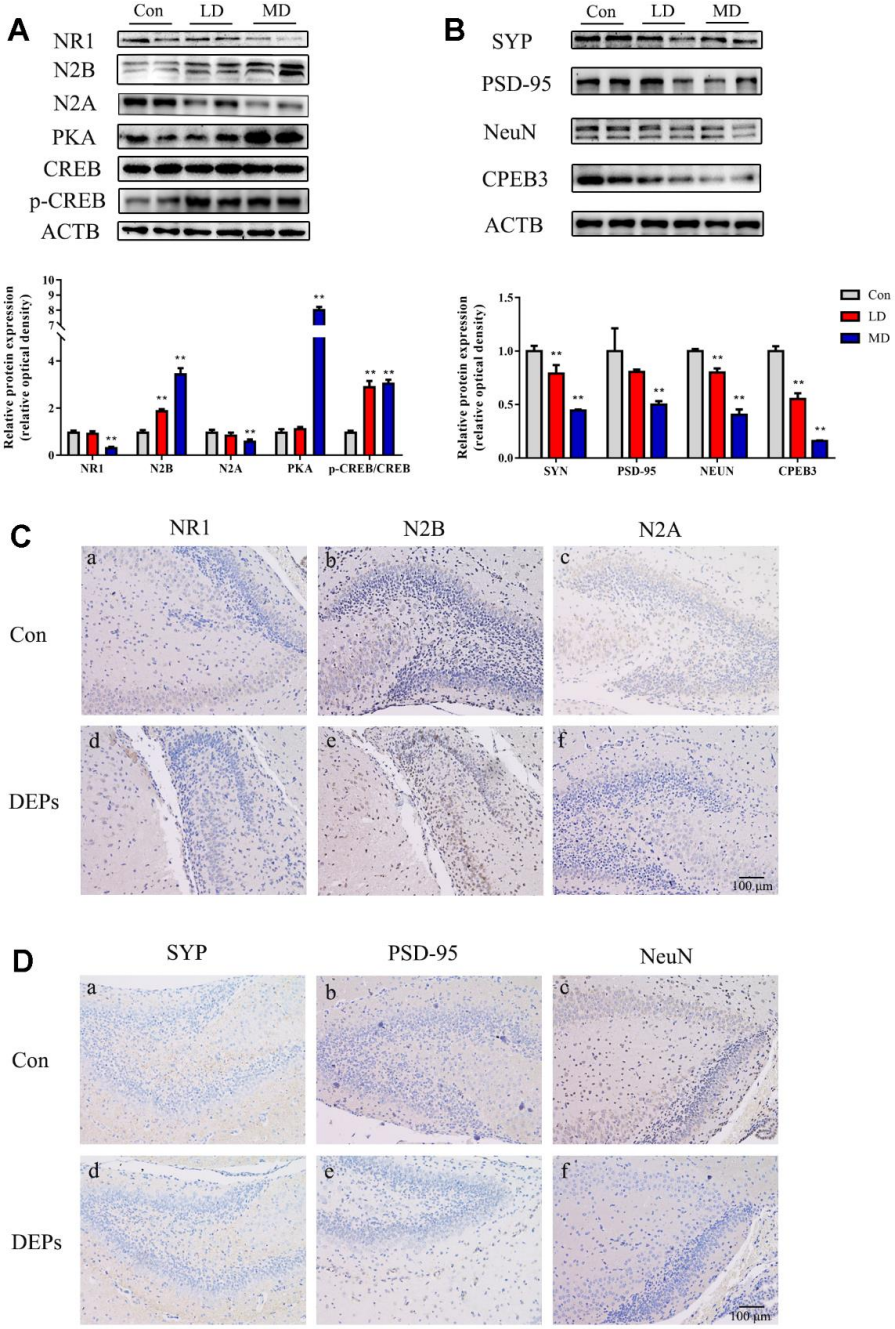
**Protein expression of NMDA/PKA/CREB and CPEB3 signaling pathway-associated genes in hippocampus samples of 7 days male offspring mice.** (**A**) Western blot analysis of p-CREB, CREB, PKA, N2A, N2B and NR1 protein in hippocampus. (**B**) Western blot analysis of CPEB3, NEUN, PSD-95 and SYN protein in hippocampus. (**C**) Immunohistochemical analysis of NR1, N2B and N2A protein in hippocampus. (**a**–**c**) Control group (CON). (**d**–**f**) DEPs treatment groups (DEPs, the MD group). (**D**) Immunohistochemical analysis of SYN, PSD-95 and NEUN protein in hippocampus. (**a**–**c**) Control group (CON). (**d**–**f**) DEPs treatment groups (DEPs, the MD group). The values shown are the mean ± SD (NAB=6, NCD=4), Compared to control; ** p < 0.01.

In this study, the Western blot analysis was conducted to examine the expression levels of NeuN, NMDA receptors (N1, N2A, and N2B), PSD95, and SYP in the hippocampus of PND7 male offspring (Figure 4A, 4B). However, due to the limited number of male offspring in the HD group, the Western blot data for the PND7 mice in the high-dose group was not available. This was done to ensure an adequate sample size for later adult behavioral and molecular experiments. The results showed that in the medium-dose (MD) group, the levels of SYP and NeuN expression were significantly lower compared with the control group (P<0.01). Additionally, the levels of N1, N2A, and PSD95 were decreased, while the NR2B expression was dramatically increased in both the low-dose (LD) and MD groups (P<0.01). To further investigate the changes in protein expression and cellular localization, immunohistochemical techniques were employed to compare the levels of these proteins between the control and MD groups (Figure 4C, 4D). The results confirmed that PSD95, SYP, NeuN, N1, and N2A subunits exhibited reduced expression in the MD group mice. The change in the cell membrane of the N2B subunit was not obvious, but an increase in the expression of the cell membrane space was observed. These findings were consistent with the Western blot results. Overall, these results suggest that exposure to DEPs throughout pregnancy leads to alterations in the expression of hippocampal synaptic transmission-related proteins and structural proteins in early male offspring (PND7).

In order to investigate whether the NMDA/PKA/CREB and CPEB3 signaling pathways are involved in the neurotoxic effects induced by DEPs, the levels of PKA, p-CREB, CREB, and CPEB3 proteins in the hippocampus were measured using Western blotting (Figure 4A, 4B). The results confirmed that the level of PKA was significantly increased in the MD group (P<0.01), but not in the LD group. Additionally, the levels of p-CREB were up-regulated, while the levels of CPEB3 were significantly down-regulated in both the LD and MD groups (P<0.01). These findings suggest that the NMDA/PKA/CREB signaling pathway may be activated, while the CPEB3 signaling pathway could be suppressed in early postnatal male offspring exposed to DEPs throughout pregnancy.

### The impact of DEP exposure throughout pregnancy on the expression of hippocampal synaptic proteins, as well as the activation of PKA/CREB and CPEB3 in mature offspring

**Figure 5:**
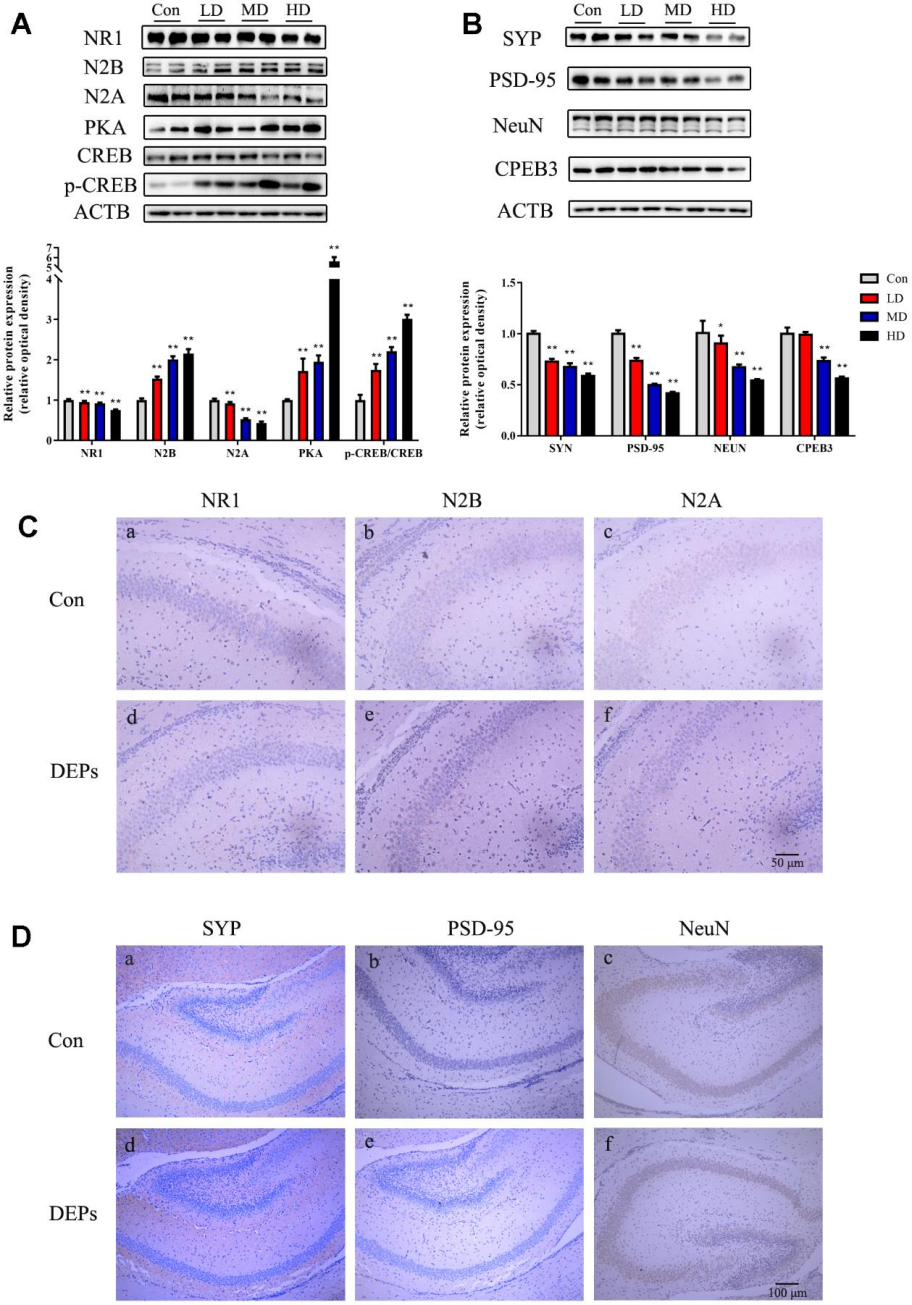
**Protein expression of NMDA/PKA/CREB and CPEB3 signaling pathway-associated genes in hippocampus samples of 56 days male offspring mice.** (**A**) Western blot analysis of p-CREB, CREB, PKA, N2A, N2B and NR1 protein in hippocampus. (**B**) Western blot analysis of CPEB3, NEUN, PSD-95 and SYN protein in hippocampus. (**C**) Immunohistochemical analysis of NR1, N2B and N2A protein in hippocampus. (**a**–**c**) Control group (CON). (**d**–**f**) DEPs treatment groups (DEPs, the MD group). (**D**) Immunohistochemical analysis of SYN, PSD-95 and NEUN protein in hippocampus. (**a**–**c**) Control group (CON). (**d**–**f**) DEPs treatment groups (DEPs, the MD group). The values shown are the mean ± SD of (N_AB=_9, N_CD=_3). Compared to control; * *p* < 0.05, ** *p* < 0.01.

The same proteins that were detected in PND7 were also found in the mature offspring of mice (Figure 5A, 5B). In the DEPs treatment groups, except for NR2B, the levels of hippocampal proteins such as SYP, NeuN, N1, N2A, and PSD95 were reduced compared to the control group (P<0.01). The results of immunohistochemical analysis in the mature offspring were similar to those in PND7 (Figure 5C, 5D). The expression of N1 and N2A subunits in the DEPs treatment groups was lower than in the control group, and there were no significant changes in the cell membrane of the N2B subunit, but the expression of the cell membrane space increased. Additionally, there was a reduction in PSD95, SYP, and NeuN protein expression in the DEPs treatment groups. These findings indicate that the hippocampal synaptic transmission function and structural related proteins changed in mature male offspring with prenatal exposure to DEPs. Furthermore, the levels of PKA and p-CREB were significantly increased in the DEPs treatment groups (P<0.01), while the levels of CPEB3 were significantly down-regulated in MD and HD groups (P<0.01) (Figure 5A, 5B). This suggests that the NMDA/PKA/CREB pathway could still be activated, but the CPEB3 signaling pathway might be suppressed in mature male offspring exposed to DEPs throughout pregnancy.

## DISCUSSION

Exposure to environmental toxicants during pregnancy could have long-lasting negative effects on neurobiology and cognitive function [27]. Therefore, we conducted an analysis of the neurotoxicity of DEPs in immature (PND7) and mature male offspring (PND56) to investigate the effects of exposure to DEPs throughout pregnancy on learning and memory abilities. Although the currently used methods of exposure, such as nasal or tracheal drip, are more realistic, there are variations in the absorption efficiency of DEPs from the nasal cavity or trachea into the bloodstream of female mice, and subsequently into the brains of the offspring through the placental barrier. These variations result in large individual errors both within and between groups, which may not accurately reflect the dose-response relationship that needs to be determined in this study. In order to minimize the error, a systemic exposure model was established in this study, using tail vein injection.

Our study focuses on investigating the potential impact on the cognitive function of male offspring mice of prenatal exposure to DEPs. The chosen method of maternal DEP exposure allows for a relatively accurate assessment of the effects of different exposure doses throughout pregnancy, thereby providing further insights into the environmental factors involved in DEP-induced neurodegeneration. Previous research has indicated that exposure to PM2.5 during pregnancy can result in low birth weight in offspring rats [28].

In our study, the dosage used did not have an impact on body weight, but it did lead to reduced performance in the Morris water maze (MWM) test, indicating impaired learning and memory. Our data demonstrated that exposure to DEPs throughout pregnancy significantly increased the time taken to find the platform and reduced the number of times the mice entered the platform area. This finding is consistent with the observations made by Alvarez et al., who reported that PM2.5 exposure impairs cognitive development in children [29]. On the contrary, previous studies have shown that exposure to DEPs in adulthood can have molecular and/or behavioral effects on the nervous system [30, 31]. The discrepancies observed among these different studies may be attributed to variations in exposure components, timing, and dosage.

The exact mechanism by which exposure to DEPs throughout pregnancy exerts toxic effects on synaptic plasticity remains unclear. Based on our results, it is hypothesized that the impairment of synaptic transmission function and synaptic structural plasticity may be a potential mechanism underlying the spatial learning and memory deficits observed following exposure to DEPs throughout pregnancy.

Indeed, hippocampal synaptic plasticity is widely recognized as the molecular and cellular neurobiological basis of memory [32]. The morphological features of synaptic plasticity in the CA1 and CA3 regions of the hippocampus include the curvature of the synaptic contact surface and the thickness of the post-synaptic density (PSD). The presynaptic vesicle protein SYP is often utilized as an indicator of synaptic density [33]. In our study, we observed that both the levels of the post-synaptic protein PSD-95 and the presynaptic protein SYP in the male offspring hippocampus which was exposed to DEPs prenatally were lower compared to the control group. This suggests an abnormal development of hippocampal synaptic structure.

The receptor activity and receptor-dependent synaptic plasticity of NMDA in the hippocampus are considered essential for spatial learning and memory [34, 35]. For the proper functioning of NMDA receptors, it is generally accepted that at least one NR1 subunit requires a glycine binding site, while each of the other NR2 subunits requires a glutamate binding site [36]. Previous studies have demonstrated that exposure to various chemical factors, such as lead and other models of nerve damage, can significantly reduce the NR1 level in hippocampal neurons [37], and NR1 gene knockout can impair spatial learning and memory in mice [38]. The NR2 subunit is composed of four subtypes, NR2A-D, with NR2A and NR2B being the primary regulatory subunits. During development, the expression of NR2A subunit and its localization at synapses gradually increase, while the NR2B subunit is crucial for neuronal formation and fetal survival [39]. The localization of NMDAR expression in synapses and extrasynaptic regions is associated with the activation of neuroprotective and apoptotic signaling cascades, respectively [16, 40]. The balance between these two localization patterns is also critical for synaptic development and synaptic plasticity [41]. In our experiment, we examined the protein expression levels of NR1, NR2A, and NR2B in the hippocampus of male offspring at postnatal day 7 (PND7) and adulthood, following exposure to different doses of DEPs throughout pregnancy. Compared to the control group, the levels of NR1 and NR2A were decreased in all DEP exposure groups, while the level of NR2B in the extracellular space was increased. This indicates a change in the subunit composition and cellular localization of NMDA receptors. These alterations may contribute to the observed deficits in cognitive function.

To further investigate the potential molecular mechanism, we measured the levels of PKA (protein kinase A) and phosphorylated CREB (cAMP response element-binding protein) in both PND7 and mature offspring. Consistent with the changes in PKA expression, the levels of p-CREB were higher in the groups exposed to DEPs throughout pregnancy compared to the control group. Furthermore, CPEB3 (cytoplasmic polyadenylation element-binding protein 3) is known to regulate the translation of important proteins involved in hippocampal long-term synaptic plasticity, including NMDAR (NR1, NR2A, and NR2B) and PSD95. CPEB3 plays a significant role in the maintenance of long-term memory, acting as a key mediator for memory consolidation and persistence [42]. In its soluble form, CPEB3 acts as an inhibitory factor in mature hippocampal neurons. However, upon synaptic stimulation, CPEB3 undergoes aggregation and transformation into an active form that promotes the translation of target proteins [43]. Our experimental results showed that, comparing with the control group, the expression of aggregated active CPEB3 decreased in the PND7 male offspring hippocampus exposed to DEPs. The expression of CPEB3 in the hippocampus of PND56 offspring also exhibited a decreasing trend, although the dose relationship was not as significant as at PND7.

Our findings indicate that maternal exposure to DEPs can result in learning and memory impairments in male offspring, which are closely associated with synaptic plasticity damage in the hippocampus. The upregulation of the PKA/CREB signaling pathway and the inhibition of CPEB3 levels in the hippocampus may contribute to the synaptic structural and functional plasticity deficits observed in male offspring following exposure to DEPs during pregnancy. It is important to note that we were unable to further test and validate this signaling pathway and the related proteins due to the challenges in simulating cross-generational toxicity effects *in vitro*. Additionally, it should be acknowledged that the method of tail vein injection may not completely replicate the actual physiological exposure experienced by humans. This could potentially amplify the impact of DEP exposure during pregnancy on the cognitive abilities of male offspring, as described in our study.

Based on our findings, we suggest that exposure to DEPs could potentially activate the PKA/CREB signaling pathway, which could in turn impact the development of synaptic structural plasticity. This is supported by the observed decrease in the expression of synaptic proteins like SYP and PSD95. Additionally, the expression and activity of CPEB3 were found to be reduced, which could further affect synaptic transmission function by inhibiting the expression of key functional proteins such as NR1, NR2A, and NR2B. Ultimately, these changes may result in irreversible damage to the cognitive abilities of adult male offspring.

## MATERIALS AND METHODS

### Animals and treatments

8-week-old C57BL/6 mice weighing 21±0.5 g were housed at the SPF Experimental Animal Center of Nantong University for 1 week for acclimation before being mated in a 2:1 cage ratio with 32 female mice and 16 male mice. The vaginal smear on the second day of cage-closure was marked as pregnancy 0 day. On the first day of pregnancy, the mother mice were randomly assigned to a control group, low-dose exposure group, middle-dose exposure group, and high-dose exposure group. To ensure experiment reproducibility and particle composition stability, commercial DEPs (the National Institute of Standards and Technology, SRM 2975; Gaithersburg, MD, USA) were used. Sterile PBS was used to prepare suspensions of different concentrations, with each suspension packed separately in 1 mL volumes. Prior to exposure, the suspensions were sonicated for 15 minutes and then heated to 37° C.

To minimize potential errors in exposure dose among offspring within the same group or even the same experimental animal, this study utilized tail vein injection as the method for DEPs exposure. This approach ensures a more accurate and consistent delivery of DEPs to the offspring brain tissue. Other exposure methods such as pharyngeal nasal drip and tracheal drip may result in variations in conversion efficiency and DEPs absorption, leading to significant errors in the actual exposure levels in the offspring brain. By employing tail vein injection, the study aims to accurately investigate the cross-generational toxicity of DEPs.

To facilitate tail vein injection, the mouse’s tail was immersed in 45° C water for 1 minute to dilate the blood vessels. A sterile 27-gauge needle syringe was prepared for the injection. Consistent with previous research reports, intravenous injection was employed to expose the mice to diesel exhaust particulate matters [44]. The injection procedure begins at the tip of the tail and progresses towards the base of the tail, with the injection site changing each time to target different blood vessels. The injection volume administered per mouse is 0.1 mL.

According to the 2012 China Environmental Air Quality Standard (GB3095-2012), the maximum allowed concentration of secondary PM2.5 for a 24-hour period is 75 μg/m^3^. To determine the exposure level of mice via the respiratory tract at this limit, we consider the breathing rate of the mice (24 ml/min) and the conversion factor for equivalent dose to the human body surface area (9.1). The calculation for the exposure level of mice through the respiratory tract is as follows: 75*24*10-6*24*60*9.1=2.35 μg. Recent research data shows that the efficiency of particulate matter in air pollution entering the brain through blood circulation may be 8 times higher than that through the nose [45]. Based on this, we used a dose equivalent to 10% (0.235 μg/mouse/time) of the maximum concentration allowed for respiratory exposure as the lowest dose (low dose group, LD) for tail vein injection. The medium dose group (MD) received 0.47 μg/mouse/time, and the high dose group (HD) received 0.94 μg/mouse/time. The tail vein injections started on the first day and were administered every other day, for a total of 10 injections. The purpose of this study is to investigate any potential neurotoxic effects of DEP exposure during pregnancy.

After birth, the newborn offspring were weighed and counted. Male offspring were specifically collected, with a breastfeeding ratio of 4 male mice per mother mouse. Subsequently, the male offspring were weighed once a week. At postnatal day 7 (PND 7), ten male mice from each group were sacrificed. Hippocampal tissues were collected from each group for tissue sections (n=4) and protein expression analysis (n=6). The remaining male offspring mice underwent a water maze experiment at PND 49. After the experiment, all mice were euthanized, and their body weight, brain weight, and hippocampus weight were measured.

### Morris water maze experiment

The Morris water maze experiment was conducted in a circular pool with a diameter of 150 cm and a depth of 22 cm. The pool was divided into four quadrants: North (N), East (E), South (S), and West (W). The water in the pool was warm and opaque, maintained at a temperature of 24 ± 2° C. A black platform with a diameter of 14 cm and a height of 21 cm was submerged in the pool. The purpose of the submerged platform was to provide a target for the mice to locate during the experiment. On the first day of the experiment, the animals were placed in the pool for a duration of 90 seconds. This initial session allowed the mice to become familiar with the maze environment, following protocols that have been previously reported [46]. The mice underwent training for five consecutive days, with each day consisting of four trials. During each trial, the mice were dropped into one of the four quadrants of the pool and allowed to swim freely until they located the submerged goal platform. Once the mice found the platform, they were required to stay on it for at least 3 seconds. If a mouse was unable to find the platform within 1 minute, it was gently guided to the platform and allowed to stay on it for 15 seconds. On the seventh day of the experiment, the platform was removed, and the mice were dropped into the pool for a duration of 60 seconds. Throughout the experiment, the swimming route, time spent in the water, and number of times the mice appeared on the platform were recorded. The SLY-WMS Morris water maze experiment system was used to conduct the test and gather the necessary data.

### Histological preparation

For morphometric analysis, fresh hippocampal tissues were harvested from the animals, fixed in 4% paraformaldehyde, and subsequently embedded in paraffin. Paraffin-embedded sections of the hippocampus, 5 μm thick, were stained with Hematoxylin-eosin (HE) for microscopic examination. All sections were examined using light microscopy.

### Immunohistochemistry analysis

The 5 μm thick paraffin-embedded hippocampal slices were mounted on slides coated with 3-aminopropyl-triethoxysilane (APES) for immunohistochemistry analysis as previous literature report [47]. After deparaffinization and drying, the paraffin sections were rinsed with NaCl solution, dehydrated, and then incubated in a solution of 3% H_2_O_2_-methanol-PBS (0.01 M, pH 7.2) for 10 minutes. Antigen retrieval was performed using trisodium citrate buffer (0.01 mol/L, pH 6.0), followed by three washes with PBS. Subsequently, the sections were treated with 10% bovine serum albumin (BSA) to block the activity of endogenous peroxidases for 1 hour. For immunostaining, the sections were incubated overnight at 4° C with primary antibodies, including neuron-specific nuclear protein (NeuN, Proteintech, 26975-1-AP), NR1 (LMAI Bio, LM-23343R), N2B (LMAI Bio, LM-0222R), N2A (Merck, AB1555P), SYP (CUSABIO, CSB-PA004200), and PSD-95 (LMAI Bio, LM-20649R). To recover the antigens, the sections were boiled in 0.01 mol/L trisodium citrate buffer (pH 6.0) and then washed three times with PBS (every 3 minutes). The sections were then stained using 3, 3’-diaminobenzidine tetrahydrochloride (Sigma, St. Louis, MO, USA). Negative controls were obtained by omitting the primary antibodies. The sections were examined and photographed using NIS-Elements BR 2.30 (40×), and subsequently counterstained with hematoxylin.

### Western blot analysis

The WB analysis is consistent with the literature [48]. The mice hippocampus was subjected to centrifugation at 12,000 g and 4° C for approximately 10 min to obtain total proteins. The concentration of the total protein was measured using the BCA Protein Assay kit (Beyotime, P0009, China). The proteins were then transferred to a polyvinylidene fluoride (PVDF) membrane after separation by sodium dodecyl sulfate polyacrylamide gel electrophoresis (Millipore Corporation, Billerica, MA, USA). The membranes were blocked with 5% nonfat milk at room temperature for 1 h, followed by incubation with specific primary antibodies. Specifically, β-actin (ACTB) was detected using (Sigma, A5316), neuron-specific nuclear protein (NeuN) with (Proteintech, 26975-1-AP), and the primary antibodies used for NR1, N2B, N2A, PKA, SYP were the same as those used in immunohistochemistry. Additionally, PSD-95 was detected with (GeneTex, GTX112846), p-CREB with (PerkinElmer, TRF0200-M), and CPEB3 with (PL0306681, PLLABS) for western blot. Following incubation with primary antibodies, the membrane was washed with TBST and treated with a peroxidase-conjugated secondary antibody (HRP-labeled goat anti-IgG, Zhongshan Biology Company, Beijing, China) diluted at 1:1000 in blocking solution for 1 hours at room temperature. Densitometry analysis of the target protein bands was performed by ImageJ software.

### Statistical analysis

The data were analyzed using GraphPad Prism software (GraphPad Software, San Diego, CA, USA) and presented as mean ± SD of independent experiments. The Wilcoxon Rank-Sum test was used for analysis of non-normally distributed data, while Student’s t-test or analysis of variance (ANOVA) was used for data with a normal distribution. A p-value of less than 0.05 was considered statistically significant.

## References

[1] Chen Q, Chen Y, Luo XS, Hong Y, Hong Z, Zhao Z, Chen J. Seasonal characteristics and health risks of PM2.5-bound organic pollutants in industrial and urban areas of a China megacity. J Environ Manage. 2019; 245:273–81. 10.1016/j.jenvman.2019.05.061 31158679

[2] Zhou Y, Guo J, Wang Z, Zhang B, Sun Z, Yun X, Zhang J. Levels and inhalation health risk of neonicotinoid insecticides in fine particulate matter (PM2.5) in urban and rural areas of China. Environ Int. 2020; 142:105822. 10.1016/j.envint.2020.105822 32497933

[3] Fussell JC, Franklin M, Green DC, Gustafsson M, Harrison RM, Hicks W, Kelly FJ, Kishta F, Miller MR, Mudway IS, Oroumiyeh F, Selley L, Wang M, Zhu Y. A Review of Road Traffic-Derived Non-Exhaust Particles: Emissions, Physicochemical Characteristics, Health Risks, and Mitigation Measures. Environ Sci Technol. 2022; 56:6813–35. 10.1021/acs.est.2c01072 35612468 PMC9178796

[4] Lawal AO, Davids LM, Marnewick JL. Diesel exhaust particles and endothelial cells dysfunction: An update. Toxicol In Vitro. 2016; 32:92–104. 10.1016/j.tiv.2015.12.015 26721178

[5] Bourdrel T, Annesi-Maesano I, Alahmad B, Maesano CN, Bind MA. The impact of outdoor air pollution on COVID-19: a review of evidence from *in vitro*, animal, and human studies. Eur Respir Rev. 2021; 30:200242. 10.1183/16000617.0242-2020 33568525 PMC7879496

[6] Maung TZ, Bishop JE, Holt E, Turner AM, Pfrang C. Indoor Air Pollution and the Health of Vulnerable Groups: A Systematic Review Focused on Particulate Matter (PM), Volatile Organic Compounds (VOCs) and Their Effects on Children and People with Pre-Existing Lung Disease. Int J Environ Res Public Health. 2022; 19:8752. 10.3390/ijerph19148752 35886604 PMC9316830

[7] Morris-Schaffer K, Merrill AK, Wong C, Jew K, Sobolewski M, Cory-Slechta DA. Limited developmental neurotoxicity from neonatal inhalation exposure to diesel exhaust particles in C57BL/6 mice. Part Fibre Toxicol. 2019; 16:1. 10.1186/s12989-018-0287-8 30612575 PMC6322252

[8] Patten KT, Valenzuela AE, Wallis C, Berg EL, Silverman JL, Bein KJ, Wexler AS, Lein PJ. The Effects of Chronic Exposure to Ambient Traffic-Related Air Pollution on Alzheimer’s Disease Phenotypes in Wildtype and Genetically Predisposed Male and Female Rats. Environ Health Perspect. 2021; 129:57005. 10.1289/EHP8905 33971107 PMC8110309

[9] Izzotti A, Spatera P, Khalid Z, Pulliero A. Importance of Punctual Monitoring to Evaluate the Health Effects of Airborne Particulate Matter. Int J Environ Res Public Health. 2022; 19:10587. 10.3390/ijerph191710587 36078301 PMC9518414

[10] Block ML, Wu X, Pei Z, Li G, Wang T, Qin L, Wilson B, Yang J, Hong JS, Veronesi B. Nanometer size diesel exhaust particles are selectively toxic to dopaminergic neurons: the role of microglia, phagocytosis, and NADPH oxidase. FASEB J. 2004; 18:1618–20. 10.1096/fj.04-1945fje 15319363

[11] O’Piela DR, Durisek GR 3rd, Escobar YH, Mackos AR, Wold LE. Particulate matter and Alzheimer’s disease: an intimate connection. Trends Mol Med. 2022; 28:770–80. 10.1016/j.molmed.2022.06.004 35840480 PMC9420776

[12] Hesterberg TW, Long CM, Lapin CA, Hamade AK, Valberg PA. Diesel exhaust particulate (DEP) and nanoparticle exposures: what do DEP human clinical studies tell us about potential human health hazards of nanoparticles? Inhal Toxicol. 2010; 22:679–94. 10.3109/08958371003758823 20462394

[13] Liu J, Yang C, Yang J, Song X, Han W, Xie M, Cheng L, Xie L, Chen H, Jiang L. Effects of early postnatal exposure to fine particulate matter on emotional and cognitive development and structural synaptic plasticity in immature and mature rats. Brain Behav. 2019; 9:e01453. 10.1002/brb3.1453 31709780 PMC6908876

[14] Zhao ZH, Zheng G, Wang T, Du KJ, Han X, Luo WJ, Shen XF, Chen JY. Low-level Gestational Lead Exposure Alters Dendritic Spine Plasticity in the Hippocampus and Reduces Learning and Memory in Rats. Sci Rep. 2018; 8:3533. 10.1038/s41598-018-21521-8 29476096 PMC5824819

[15] Stark SM, Kirwan CB, Stark CE. Mnemonic Similarity Task: A Tool for Assessing Hippocampal Integrity. Trends Cogn Sci. 2019; 23:938–51. 10.1016/j.tics.2019.08.003 31597601 PMC6991464

[16] Sun D, Li S, Huang H, Xu L. Neurotoxicity of melittin: Role of mitochondrial oxidative phosphorylation system in synaptic plasticity dysfunction. Toxicology. 2023; 497–8:153628. 10.1016/j.tox.2023.153628 37678661

[17] Fricker M, Tolkovsky AM, Borutaite V, Coleman M, Brown GC. Neuronal Cell Death. Physiol Rev. 2018; 98:813–80. 10.1152/physrev.00011.2017 29488822 PMC5966715

[18] Zhang T, Zheng X, Wang X, Zhao H, Wang T, Zhang H, Li W, Shen H, Yu L. Maternal Exposure to PM2.5 during Pregnancy Induces Impaired Development of Cerebral Cortex in Mice Offspring. Int J Mol Sci. 2018; 19:257. 10.3390/ijms19010257 29337904 PMC5796203

[19] Zheng Y, Ding W, Zhang T, Zhao Z, Wang R, Li Z, Yu S, Li J, Zhao X, Wu Q. Antimony-induced astrocyte activation via mitogen-activated protein kinase activation-dependent CREB phosphorylation. Toxicol Lett. 2021; 352:9–16. 10.1016/j.toxlet.2021.09.006 34571074

[20] Zhu G, Liu Y, Zhi Y, Jin Y, Li J, Shi W, Liu Y, Han Y, Yu S, Jiang J, Zhao X. PKA- and Ca2+-dependent p38 MAPK/CREB activation protects against manganese-mediated neuronal apoptosis. Toxicol Lett. 2019; 309:10–9. 10.1016/j.toxlet.2019.04.004 30951808

[21] Malenka RC, Nicoll RA. NMDA-receptor-dependent synaptic plasticity: multiple forms and mechanisms. Trends Neurosci. 1993; 16:521–7. 10.1016/0166-2236(93)90197-t 7509523

[22] Shah FA, Liu G, Al Kury LT, Zeb A, Abbas M, Li T, Yang X, Liu F, Jiang Y, Li S, Koh PO. Melatonin Protects MCAO-Induced Neuronal Loss via NR2A Mediated Prosurvival Pathways. Front Pharmacol. 2019; 10:297. 10.3389/fphar.2019.00297 31024297 PMC6461025

[23] Benske TM, Mu TW, Wang YJ. Protein quality control of N-methyl-D-aspartate receptors. Front Cell Neurosci. 2022; 16:907560. 10.3389/fncel.2022.907560 35936491 PMC9352929

[24] Liu J, Chang L, Roselli F, Almeida OF, Gao X, Wang X, Yew DT, Wu Y. Amyloid-β induces caspase-dependent loss of PSD-95 and synaptophysin through NMDA receptors. J Alzheimers Dis. 2010; 22:541–56. 10.3233/JAD-2010-100948 20847396

[25] Yao M, Meng M, Yang X, Wang S, Zhang H, Zhang F, Shi L, Zhang Y, Zhang X, Xu Z. POSH regulates assembly of the NMDAR/PSD-95/Shank complex and synaptic function. Cell Rep. 2022; 39:110642. 10.1016/j.celrep.2022.110642 35385725

[26] Qu WR, Sun QH, Liu QQ, Jin HJ, Cui RJ, Yang W, Song B, Li BJ. Role of CPEB3 protein in learning and memory: new insights from synaptic plasticity. Aging (Albany NY). 2020; 12:15169–82. 10.18632/aging.103404 32619199 PMC7425470

[27] Heyer DB, Meredith RM. Environmental toxicology: Sensitive periods of development and neurodevelopmental disorders. Neurotoxicology. 2017; 58:23–41. 10.1016/j.neuro.2016.10.017 27825840

[28] Dang S, Ding D, Lu Y, Su Q, Lin T, Zhang X, Zhang H, Wang X, Tan H, Zhu Z, Li H. PM2.5 exposure during pregnancy induces hypermethylation of estrogen receptor promoter region in rat uterus and declines offspring birth weights. Environ Pollut. 2018; 243:851–61. 10.1016/j.envpol.2018.09.065 30245447

[29] Alvarez-Pedrerol M, Rivas I, López-Vicente M, Suades-González E, Donaire-Gonzalez D, Cirach M, de Castro M, Esnaola M, Basagaña X, Dadvand P, Nieuwenhuijsen M, Sunyer J. Impact of commuting exposure to traffic-related air pollution on cognitive development in children walking to school. Environ Pollut. 2017; 231:837–44. 10.1016/j.envpol.2017.08.075 28866425

[30] Shkirkova K, Lamorie-Foote K, Zhang N, Li A, Diaz A, Liu Q, Thorwald MA, Godoy-Lugo JA, Ge B, D’Agostino C, Zhang Z, Mack WJ, Sioutas C, et al. Neurotoxicity of Diesel Exhaust Particles. J Alzheimers Dis. 2022; 89:1263–78. 10.3233/JAD-220493 36031897

[31] Tobwala S, Zhang X, Zheng Y, Wang HJ, Banks WA, Ercal N. Disruption of the integrity and function of brain microvascular endothelial cells in culture by exposure to diesel engine exhaust particles. Toxicol Lett. 2013; 220:1–7. 10.1016/j.toxlet.2013.03.023 23542817 PMC6007876

[32] Cornell J, Salinas S, Huang HY, Zhou M. Microglia regulation of synaptic plasticity and learning and memory. Neural Regen Res. 2022; 17:705–16. 10.4103/1673-5374.322423 34472455 PMC8530121

[33] Shi Y, Nan C, Yan Z, Liu L, Zhou J, Zhao Z, Li D. Synaptic Plasticity of Human Umbilical Cord Mesenchymal Stem Cell Differentiating into Neuron-like Cells *In Vitro* Induced by Edaravone. Stem Cells Int. 2018; 2018:5304279. 10.1155/2018/5304279 30510585 PMC6230402

[34] Hansen KB, Yi F, Perszyk RE, Furukawa H, Wollmuth LP, Gibb AJ, Traynelis SF. Structure, function, and allosteric modulation of NMDA receptors. J Gen Physiol. 2018; 150:1081–105. 10.1085/jgp.201812032 30037851 PMC6080888

[35] Shi L, Adams MM, Long A, Carter CC, Bennett C, Sonntag WE, Nicolle MM, Robbins M, D’Agostino R, Brunso-Bechtold JK. Spatial learning and memory deficits after whole-brain irradiation are associated with changes in NMDA receptor subunits in the hippocampus. Radiat Res. 2006; 166:892–9. 10.1667/RR0588.1 17149974

[36] Staley EM, Jamy R, Phan AQ, Figge DA, Pham HP. N-Methyl-d-aspartate Receptor Antibody Encephalitis: A Concise Review of the Disorder, Diagnosis, and Management. ACS Chem Neurosci. 2019; 10:132–42. 10.1021/acschemneuro.8b00304 30134661

[37] Shimizu E, Tang YP, Rampon C, Tsien JZ. NMDA receptor-dependent synaptic reinforcement as a crucial process for memory consolidation. Science. 2000; 290:1170–4. 10.1126/science.290.5494.1170 11073458

[38] Liu L, Wong TP, Pozza MF, Lingenhoehl K, Wang Y, Sheng M, Auberson YP, Wang YT. Role of NMDA receptor subtypes in governing the direction of hippocampal synaptic plasticity. Science. 2004; 304:1021–4. 10.1126/science.1096615 15143284

[39] Tovar KR, Westbrook GL. The incorporation of NMDA receptors with a distinct subunit composition at nascent hippocampal synapses *in vitro*. J Neurosci. 1999; 19:4180–8. 10.1523/JNEUROSCI.19-10-04180.1999 10234045 PMC6782704

[40] Yan J, Bading H. The Disruption of NMDAR/TRPM4 Death Signaling with TwinF Interface Inhibitors: A New Pharmacological Principle for Neuroprotection. Pharmaceuticals (Basel). 2023; 16:1085. 10.3390/ph16081085 37631001 PMC10458786

[41] Liu J, Chang L, Song Y, Li H, Wu Y. The Role of NMDA Receptors in Alzheimer’s Disease. Front Neurosci. 2019; 13:43. 10.3389/fnins.2019.00043 30800052 PMC6375899

[42] Chao HW, Tsai LY, Lu YL, Lin PY, Huang WH, Chou HJ, Lu WH, Lin HC, Lee PT, Huang YS. Deletion of CPEB3 enhances hippocampus-dependent memory via increasing expressions of PSD95 and NMDA receptors. J Neurosci. 2013; 33:17008–22. 10.1523/JNEUROSCI.3043-13.2013 24155305 PMC6618447

[43] Drisaldi B, Colnaghi L, Fioriti L, Rao N, Myers C, Snyder AM, Metzger DJ, Tarasoff J, Konstantinov E, Fraser PE, Manley JL, Kandel ER. SUMOylation Is an Inhibitory Constraint that Regulates the Prion-like Aggregation and Activity of CPEB3. Cell Rep. 2015; 11:1694–702. 10.1016/j.celrep.2015.04.061 26074071 PMC5477225

[44] Hubner EK, Lechler C, Rösner TN, Kohnke-Ertel B, Schmid RM, Ehmer U. Constitutive and Inducible Systems for Genetic *In Vivo* Modification of Mouse Hepatocytes Using Hydrodynamic Tail Vein Injection. J Vis Exp. 2018:56613. 10.3791/56613 29443066 PMC5912325

[45] Obermair A, Binder M, Barrada M, Bancher-Todesca D, Asseryanis E, Kubista E. Onycholysis in patients treated with docetaxel. Ann Oncol. 1998; 9:230–1. 10.1023/a:1008218824342 9553674

[46] Fu L, Liu C, Chen L, Lv Y, Meng G, Hu M, Long Y, Hong H, Tang S. Protective Effects of 1-Methylnicotinamide on Aβ1-42-Induced Cognitive Deficits, Neuroinflammation and Apoptosis in Mice. J Neuroimmune Pharmacol. 2019; 14:401–12. 10.1007/s11481-018-09830-1 30635816

[47] Zhao X, Wu Y, Li J, Li D, Jin Y, Zhu P, Liu Y, Zhuang Y, Yu S, Cao W, Wei H, Wang X, Han Y, Chen G. JNK activation-mediated nuclear SIRT1 protein suppression contributes to silica nanoparticle-induced pulmonary damage via p53 acetylation and cytoplasmic localisation. Toxicology. 2019; 423:42–53. 10.1016/j.tox.2019.05.003 31082419

[48] Qiu L, Wang H, Dong T, Huang J, Li T, Ren H, Wang X, Qu J, Wang S. Perfluorooctane sulfonate (PFOS) disrupts testosterone biosynthesis via CREB/CRTC2/StAR signaling pathway in Leydig cells. Toxicology. 2021; 449:152663. 10.1016/j.tox.2020.152663 33359577

